# Platelet-Rich Plasma: A Promising Regenerative Therapy in Gynecological Disorders

**DOI:** 10.7759/cureus.28998

**Published:** 2022-09-10

**Authors:** Jerin Varghese, Neema Acharya

**Affiliations:** 1 College of Medicine, Jawaharlal Nehru Medical College, Datta Meghe Institute of Medical Sciences, Wardha, IND; 2 Department of Obstetrics and Gynaecology, Jawaharlal Nehru Medical College, Datta Meghe Institute of Medical Sciences, Wardha, IND

**Keywords:** prp, disorders, regenerative medicine therapies, gynaecology, platelet-rich plasma/ prp

## Abstract

Platelet-rich plasma (PRP) could be understood as a special preparation of plasma in which the concentration of platelet is immensely high. This rationale for plasma use has been in the medical science for many years with plenty of success in various fields where it was inculcated, bringing dramatically favorable and better outcomes in terms of disease management and prognosis. PRP has been widely used in orthopedics from the very beginning, but in the past few years its use has been extended to other fields too, such as obstetrics and gynecology. From the very onset of the introduction of platelet-rich plasma in gynecology, there had been constant research being carried out all around the globe in order to scientifically prove and confirm its exact role in the management of gynecological problems. Regenerative medicine in gynecology was among the first areas where the platelet-rich plasma was implemented and has substantially given great results, which encouraged further extensive research to be carried out in other spectrums of gynecology. The implications of such great struggles ultimately gave way to evidence suggesting the importance of platelet-rich plasma in managing gynecological disorders like Asherman’s syndrome, urinary incontinence, genital fistulas, thin endometrium, etc. This review article collectively summarizes the various use of platelet-rich plasma in gynecology.

## Introduction and background

Platelet-rich plasma (PRP) may be understood as an autologous plasma preparation that is enriched with an increased concentration of platelet when compared to those present in whole blood [1]. The immense potential of platelet-rich plasma is often not made use of, as this particular entity is seldom understood in depth as it should be. Research is being conducted all around the globe to understand the true potential of platelet-rich plasma, and undoubtedly a deeper knowledge of the same could be a solution to many problems in medical science, as this would pave the way for advanced and affordable therapeutic management strategies. Platelet-rich plasma (PRP) is obtained by the centrifugation of whole blood [2]. PRP contains growth factors and bioactive proteins that positively aid in the healing of ligament, bone, tendon and muscle [3]. It has been used in sports medicine to a good extent because of its effects on the musculoskeletal system. PRP has been helpful in tackling medical conditions in sports like muscle strains, Achilles tendon, lateral epicondylitis, ligament strains, rotator cuff tears, etc. [4].The incorporation of PRP into the hydrogel, which on biomineralization, could significantly accelerate the generation of bone [5]. In patients with androgenic alopecia, the use of PRP has the potential for hair restoration [6]. Apart from the increment in hair growth, the quality and density of the hair are also increased with its use [7]. It is also used in plastic facial surgeries because of its potential in the healing of wounds [8]. PRP, when combined with autologous fat grafting, laser therapies, dermal fillers, and microneedling, have been found to have synergistic effects that lead to better aesthetic outcomes apart from their widespread applications in dermatology like the areas of acne scars, striae distensae, skin rejuvenation, dermal augmentation, hair restoration, etc. [9]. PRP is a potential candidate for the regeneration of damaged tissues, including the liver and the dental pulp [10]. Autologous PRP could be developed easily and is an effective surgical adjunct, which is proven to be helpful in accelerating postsurgical healing in oral surgeries and periodontics [11]. The extensive use of the platelet-rich plasma in gynecology is hopefully expected to give good positive results, as was seen in musculoskeletal system and skin regeneration.

The abundant use of PRP in regenerative medicine has provoked researchers all around the world to apply its potential in other fields of medical science too. These efforts were carried out in obstetrics and gynecology as well, and the outcome was a wide range of applications in the domain of reproductive medicine, particularly in Asherman’s syndrome, cases of thin endometrium, urinary incontinence, recurrent genitourinary fistula auxiliary treatment, etc. [12]. Although this requires more research, with the little information that is available now from the few research conducted, there is a good hope that in the near future, platelet-rich plasma could resolve various challenges that are currently being faced in obstetrics and gynecology. Some of its achievements in gynecology and obstetrics are a decrease in the FSH levels, increased endometrial thickness, increased anti-Mullerian hormone (AMH), etc. [13]. If the potential of PRP is utilized, then women who suffer from premature ovarian insufficiencies, poor ovarian reserve, and even early menopause cases where they are trying to conceive using their own oocytes may find this helpful [14]. The benefits of the use of PRP over conventional management strategies in obstetrics and gynecology include minimally invasive procedures, low cost, easy availability, lesser adverse effects, etc., but the issue that is quite prominent is that there is as such, no standardized concentration of PRP that could effectively resolve gynecological problems in one go. PRP is concerned that they shouldn’t be used in coagulation disorder patients [15]. The use of platelet-rich plasma in patients with coagulation disorders would worsen their general condition at a very rapid pace, thus, should never be used in these patients. There are other conditions too, where it is prudent to avoid its use as a management modality, such as cases of active infections or patients under NSAIDs; particularly with respect to gynecology, it should never be prescribed to pregnant women and lactating mothers [16]. Stress urinary incontinence and overactive bladder are two gynecological entities that affect a vast majority of women, and the good part is that treatment with PRP had a profound impact on these disorders [12].

## Review

Although platelet-rich plasma (PRP) therapies have a huge potential to revolutionize the management of various gynecological conditions at this point in time, only a very less fraction of the same is made a reality. The continuing research in the field of gynecology has hope for the involvement of more of this gracious and effective management strategy and, for the time being, more research is to be conducted to deepen the understanding of the effects of PRP on the diseases it is being tried out. Here, in this review article, some of the widely promising and practically applicable aspects of the gynecological manifestations would be dealt with.

An intact endometrium is one of the main prerequisites for the implantation to take place. Much of the implantation-related pregnancy issues do take place as a result of the disparities in the endometrial layer. A proper endometrium is a must for safe implantation. Platelet-rich plasma interventions have been shown to successfully figure out several issues of the endometrium and thus have proved beneficial to many women suffering from endometrial problems. Platelet-rich plasma increased the receptivity of the endometrium and thereby led to a rise in the rate of implantation. In patients with thin endometrium, platelet-rich plasma is effective in the growth of endometrium as it restores the structure of the endometrium and decreases fibrosis. Their role in the management of Asherman’s syndrome is also phenomenal. It aids in the management of patients with endometrial difficulties that are a result of associated chronic systemic diseases and thus has emerged as a golden ray of hope even in these low prognosis patients. Various studies have proved its effectiveness in endometrial abnormalities [17-20] (Table 1).

**Table 1:** Effectiveness of platelet-rich plasma in endometrial abnormalities. FET: Frozen embryo transfer, PCOS: Polycystic ovary syndrome, TB: Tuberculosis, DOR: Decreased ovarian reserve, CPR: Cardiopulmonary resuscitation

TITLE OF RESEARCH	IMPLICATION
Platelet-rich plasma as an adjuvant in the endometrial preparation of patients with refractory endometrium	Platelet-rich plasma provided an increase in endometrial receptivity and a consequent increase in implantation rates
Treatment of thin endometrium with autologous platelet-rich plasma: a pilot study	PRP was effective for endometrial growth in patients with thin endometrium
Intrauterine Infusion of Human Platelet-Rich Plasma Improves Endometrial Regeneration and Pregnancy Outcomes in a Murine Model of Asherman's Syndrome	The infusion of intrauterine human PRP restores endometrial structure and decreases fibrosis in a well-established AS murine model
Autologous platelet-rich plasma optimizes endometrial thickness and pregnancy outcomes in women with refractory thin endometrium of varied aetiology during fresh and frozen-thawed embryo transfer cycles	PRP significantly enhances endometrial thickness during fresh and FET cycles associated with TB, PCOS and DOR, thus improving the CPR and LBR in these low prognosis patients

Platelet-rich plasma (PRP) has promising positive impacts on the growth of follicles alongside endometrium and thus has become a good alternative for older women who possess low follicular reserve and unresponsive endometrium seeking motherhood [21]. The improvement in the microenvironment of ovaries, along with the provision of growth factors for germline stem cells of the ovaries makes platelet-rich plasma a potential management strategy for low reserves of the ovaries [22]. The injection (intra-ovarian) of calcium gluconate-activated autologous platelet-rich plasma is likely to improve the functions of the ovaries after a span of two months in 38 to 46 years age group women [23]. The advantage of the ovaries being a massively angiogenic organ is that it could be expected that neoangiogenesis in ovarian tissues could be brought about by angiogenic factors that are derived from the platelet-rich plasma, which, in turn, makes room for the reactivation and regeneration of tissue [24].

In the autologous transplantation of ovaries, the angiogenic potential of platelet-rich plasma is made handy [25]. Damages to the ovaries that occur as a result of torsion-related ischemia would be reduced to a good extent by the administration of platelet-rich plasma intraperitoneally [26]. Oxidative stress-induced injuries are prevented with the help of platelet-rich plasma as it increases vascular endothelial growth factor (VEGF) and a few nuclear factors that aid in angiogenesis [27]. Even though platelet-rich plasma is made useful in various ovary-related problems but more research would help add more gynecological problems to the list that PRP could resolve. Platelet-rich plasma is serving as a good management strategy in tackling many of the urinary problems of patients attending gynecology clinics. It has a profound effect as a supportive treatment modality in case of recurrent vesicovaginal fistulas. Disorders of the urinary tract and difficulty in urination were seldom complained by patients in whom platelet-rich plasma was used as adjuvant therapy for the treatment of recurrent vesicovaginal fistula [28]. PRP is also found to be of use in the management of cystoceles. Considering the complications of cystoceles like mesh complications, platelet-rich plasma may serve as a good option to prevent the recurrence of cystoceles [29].

Platelet-rich plasma is useful in cases of stress urinary incontinence (SUI) as PRP contains several growth factors that help in the reconstruction of the damaged ligament in, i.e., pubourethral ligament [30]. Urethral sphincter injection of platelet-rich plasma is a minimally invasive, safe, and effective treatment modality in cases of postprostatectomy urinary incontinence with considerable urodynamic and clinical evidence [31]. Platelet-rich plasma helps not only in the management of urogynaecological problems but is handy in the diagnosis of certain conditions like painful bladder syndrome/interstitial cystitis. It has the potential to act as a modulator of urothelial repair, and this could be made useful in painful bladder syndromes [32]. Various research in the past couple of years is clearly suggestive of the fact that intravesical platelet-rich plasma reduces chronic inflammation in bladder pain syndrome/interstitial cystitis (BPS/IC) as they could improve regeneration of the urothelium [33].

There is clinical evidence that the injections of platelet-rich plasma could substantially decrease the urinary inflammatory proteins in interstitial cystitis/bladder pain syndrome (IC/BPS) and thus aids in the improvement of symptoms [34]. A study demonstrated that autologous platelet-rich plasma intravesical injections helped to improve interstitial cystitis as it safely decreases MMP-13, urinary NGF, and levels of VEGF [35]. The instillation of intravesical PRP has been found to increase the mitotic index in cyclophosphamide and saline groups, along with aiding in the decrement of bleeding macroscopically [36]. Considering the outcomes of multiple types of research, we could come to an inference that the intravesical injection of platelet-rich plasma could potentially act as an effective and safe treatment option in cases of bladder pain syndrome by its multiple actions that aid in tissue regeneration, wound healing and modulation of the immunity [37].

## Conclusions

After having a careful review of the various research works on the use of platelet-rich plasma in gynecology, published on internationally recognized scientific platforms, various valuable insights were obtained, which have the potential to bring about a great revolution in obstetrics and gynecology. Starting from very minor ailments and spanning to some of the most chronic forms of gynecological issues, platelet-rich plasma is undoubtedly a worthy candidate for the management of these problems. The wide spectrum of its usage has made various gynecological issues such as thin endometrium, recurrent genital fistulas, ovarian abnormalities, Asherman’s syndrome, urinary stress incontinence, etc., effectively managed. Platelet-rich plasma has been scientifically proven to increase the thickness of the endometrium. Thereby aiding implantation in women as implantation wouldn’t have been otherwise possible in these females because of their thin endometrium. Those women who were suffering from ovary-related issues benefited when platelet-rich plasma was administered to them. The role of platelet-rich plasma in the management of urinary complaints is phenomenal, and thus the implementation of PRP substantially improves the symptoms in these women. From the analysis of the effects of platelet-rich plasma in gynecology which is currently available, there is no doubt that more research would aid in discovering the hidden potentials of platelet-rich plasma in the management of many more of the issues encountered in gynecology.
